# Increased Salt Intake Decreases Diet-Induced Thermogenesis in Healthy Volunteers: A Randomized Placebo-Controlled Study

**DOI:** 10.3390/nu14020253

**Published:** 2022-01-07

**Authors:** Anja Mähler, Samuel Klamer, András Maifeld, Hendrik Bartolomaeus, Lajos Markó, Chia-Yu Chen, Sofia K. Forslund, Michael Boschmann, Dominik N. Müller, Nicola Wilck

**Affiliations:** 1 Experimental and Clinical Research Center (ECRC), A Cooperation between Charité-Universitätsmedizin Berlin and Max-Delbrück-Center for Molecular Medicine, 13125 Berlin, Germany; samuel.klamer@charite.de (S.K.); andras.maifeld@mdc-berlin.de (A.M.); hendrik.bartolomaeus@charite.de (H.B.); lajos.marko@charite.de (L.M.); chia-yu.chen@mdc-berlin.de (C.-Y.C.); sofia.forslund@mdc-berlin.de (S.K.F.); michael.boschmann@charite.de (M.B.); dominik.mueller@mdc-berlin.de (D.N.M.); nicola.wilck@charite.de (N.W.); 2 Charité–Universitätsmedizin Berlin, Corporate Member of Freie Universität Berlin, Humboldt Universität zu Berlin, and Berlin Institute of Health, 10117 Berlin, Germany; 3 Max-Delbrück-Center for Molecular Medicine (MDC) in the Helmholtz Association, 13125 Berlin, Germany; 4 DZHK (German Centre for Cardiovascular Research), Partner Site Berlin, 10785 Berlin, Germany; 5 Department of Nephrology and Internal Intensive Care Medicine, Charité-Universitätsmedizin Berlin, Corporate Member of Freie Universität Berlin and Humboldt-Universität zu Berlin, 10117 Berlin, Germany

**Keywords:** salt challenge, indirect calorimetry, blood pressure, body composition, food intake, 24 h urine analysis

## Abstract

High salt intake ranks among the most important risk factors for noncommunicable diseases. Western diets, which are typically high in salt, are associated with a high prevalence of obesity. High salt is thought to be a potential risk factor for obesity independent of energy intake, although the underlying mechanisms are insufficiently understood. A high salt diet could influence energy expenditure (EE), specifically diet-induced thermogenesis (DIT), which accounts for about 10% of total EE. We aimed to investigate the influence of high salt on DIT. In a randomized, double-blind, placebo-controlled, parallel-group study, 40 healthy subjects received either 6 g/d salt (NaCl) or placebo in capsules over 2 weeks. Before and after the intervention, resting EE, DIT, body composition, food intake, 24 h urine analysis, and blood pressure were obtained. EE was measured by indirect calorimetry after a 12 h overnight fast and a standardized 440 kcal meal. Thirty-eight subjects completed the study. Salt intake from foods was 6 g/d in both groups, resulting in a total salt intake of 12 g/d in the salt group and 6 g/d in the placebo group. Urine sodium increased by 2.29 g/d (*p* < 0.0001) in the salt group, indicating overall compliance. The change in DIT differed significantly between groups (placebo vs. salt, *p* = 0.023). DIT decreased by 1.3% in the salt group (*p* = 0.048), but increased by 0.6% in the placebo group (*NS*). Substrate oxidation indicated by respiratory exchange ratio, body composition, resting blood pressure, fluid intake, hydration, and urine volume did not change significantly in either group. A moderate short-term increase in salt intake decreased DIT after a standardized meal. This effect could at least partially contribute to the observed weight gain in populations consuming a Western diet high in salt.

## 1. Introduction

Salt (sodium chloride, NaCl) is an essential nutrient, of which too high as well as too low intakes can cause adverse health outcomes [1]. Salt intakes recorded in several studies are typically higher than recommended by international and national organizations. The World Health Organization (WHO) and the German Nutrition Society (DGE) recommend 5 and 6 g/d, respectively [2,3]. However, estimated actual intakes in Germany are 8.4 g/d in women and 10.0 g/d in men [4].

A diet high in salt ranks among the most important risk factors for noncommunicable diseases [5]. It is well established that high salt intake can promote hypertension [5], which contributes to the cardiovascular disease burden. In 2017, 3 million deaths and 70 million disability-adjusted life-years globally were attributable to high sodium intake. About 2.7 million of these deaths resulted from cardiovascular diseases [6].

High salt intake has also been implicated in the development of obesity. Previously, it was postulated that salty foods increase thirst leading to higher consumption of sugary beverages, especially in children [7,8]. However, more recent evidence suggests that salt intake might be a risk factor for obesity, independent of energy intake. A cross-sectional study in the U.K. found that 1 g/d increase in salt intake was associated with a 26% higher risk for obesity in adults [9]. The INTERMAP study showed a similar association for Japan (21%), China (4%), the U.K. (29%), and the U.S. (24%) [10].

Obesity, i.e., excessive body fat accumulation, is caused by an imbalance of energy intake and energy expenditure (EE). Total EE is comprised of resting EE, diet-induced thermogenesis (DIT), and activity EE. In a pilot study in eight healthy men, we found that resting EE was unchanged after 2 weeks of increased salt intake. However, DIT was markedly decreased. Based on this finding, we designed a randomized, placebo-controlled study to test the hypothesis that a moderate 6 g/d increase in salt intake over 2 weeks decreases DIT compared to placebo.

## 2. Materials and Methods

### 2.1. Study Design

This was a randomized, double-blind, placebo-controlled, parallel group study (ClinicalTrials.gov; NCT03024567). It was carried out at the Experimental & Clinical Research Center of Charité-Universitätsmedizin in Berlin, from January to December 2017.

### 2.2. Participants

**Figure 1:**
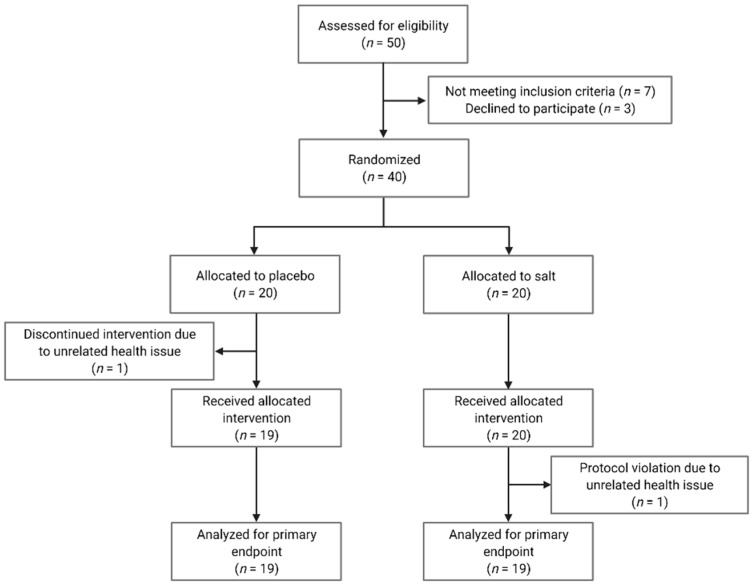
Consolidated Standards of Reporting Trials (CONSORT) flow diagram.

We screened 50 subjects in our Clinical Research Unit of which 40 were included in the study (Figure 1). Subjects were enrolled between January and December 2017. We included healthy men and women, aged between 18 and 50 years, with a body mass index between 18.5 and 29.9 kg/m^2^. Key exclusion criteria were heart, lung, liver, and kidney diseases in need of treatment; all autoimmune diseases; current or chronic infection; habitual use of probiotics or dietary supplements; pregnancy; and lactation. All participants were non-smokers. The institutional review board of Charité-Universitätsmedizin Berlin approved the study protocol (EA1/340/15) and we obtained written informed consent from all participants prior to study entry. There were no changes to methods after study commencement.

### 2.3. Intervention

Subjects received enteric-coated capsules containing either 0.75 g of sodium chloride/capsule or a similar amount of porcine gelatine (Gelatina Alba A, identically appearing placebo). The dose regimen during the 2-week intervention was 3–2–3 capsules in the morning, at noon, and in the evening, respectively. This regimen increased salt intake by 6 g/d. Capsules were produced according to good manufacturing practice by Hubertus Apotheke (Am Salzufer, Berlin, Germany). This intervention allowed both a highly standardized increase in salt intake as well as complete blinding (no sensory perception of salt). Apart from capsule intake, we asked participants to maintain their habitual diet during the intervention period.

### 2.4. Randomization

Randomization was based on 2 computer-generated lists, 1 for men and 1 for women (20 subjects per list, block size 4). These lists were used by the manufacturer to provide the study center with sequentially numbered capsule containers, thereby allowing complete blinding. An external person not involved in the study received opaque envelopes, which contained the treatment allocation. All people involved in the study, i.e., participants, study personnel, data collectors, and outcome assessors were blinded to the treatment allocation.

### 2.5. Outcome Measures

Outcome measures were predetermined and did not change during or after the study. The primary outcome measure was DIT within 4 h after a standardized meal (% energy intake), after 2 weeks of salt vs. placebo, assessed by indirect calorimetry. Secondary outcome measures were body composition, dietary sodium intake, urine sodium excretion, blood pressure, and pulse wave velocity.

### 2.6. Study Center Assessments

We assessed participants before and after the 2-week intervention. Assessments started in the morning, after a 12 h overnight fast. Participants were asked to refrain from caffeine, alcohol and vigorous exercise for 24 h before the assessments.

Body composition was determined by bioelectrical impedance analysis (Biacorpus RX 4000, MEDI CAL Healthcare GmbH, Karlsruhe, Germany). We measured oxygen (O_2_) consumption and carbon dioxide (CO_2_) production by indirect calorimetry with the canopy method (Quark RMR, COSMED, Italy). The software of the calorimetry device provided VO_2_ and VCO_2_ values for each minute of the measurement period. We calculated means over 15 min periods for both measures. Single values that deviated more than 10% from this mean were excluded from the analysis. The revised 15 min means were used to calculate RER (VCO_2_/VO_2_) and EE according to the formula by Steiniger et al. [11].

EE (kJ/min) = 16.18 × VO_2_ (L/min) + 5.02 × VCO_2_ (L/min) − 5.99 × N_ex_ (1)

Nitrogen excretion was assumed to be constant at 5.56 mg/min. DIT was calculated by the following formulas:

Total DIT over 4 h (kJ) = Total EE (kJ/min) − resting EE (kJ/min) × 240 min(2)

Percent DIT of meal (%) = DIT (kJ)/1845 kJ energy content meal × 100(3)

Measurements were conducted in the supine position after a 30 min resting period. After resting EE measurements (30 min), we provided participants with a standardized breakfast (bread, butter, curd, cheese, and cucumber) prepared by a dietician. This meal contained 441 kcal with 50, 30, and 20% of energy from carbohydrates, fats, and proteins, respectively. In addition, participants received 150 mL of still mineral water with low sodium content. All participants had to finish their meal within 15 min, which was possible for everybody. Afterwards, we continued calorimetry for 4 h with 15 min breaks at the beginning of each hour. During this time, no additional food or water intake was allowed.

During these 6 h, blood pressure and pulse wave velocity were measured automatically every 15 min with an upper arm cuff (Mobil-o-Graph, I.E.M. GmbH, Stolberg, Germany). The appropriate cuff size was determined by trained personnel and the cuff was placed on the non-dominant arm.

### 2.7. At-Home Assessments

On the three consecutive days before study center assessments, participants recorded their dietary intake in food records and collected three 24 h urine specimens. To analyze energy, macro- and micronutrient, and fluid intake, a validated software based on the German Nutrient Database was used (OptiDiet version 5.0.2.010). Urine was analyzed by an accredited laboratory according to international standards.

### 2.8. Sample Size Calculation and Data Analysis

This was a confirmatory study, based on our previously recorded pilot data in healthy men, also subjected to a 6 g/d increase in salt intake for 2 weeks (ClinicalTrials.gov; NCT02509962, EA1/138/15). In this pilot study, we observed a reduction of DIT within 4 h after the test meal (described above) from 16% to 11%. Based on this effect size of 5% (standardized effect size 0.95, alpha error 0.05, beta error 0.10), we calculated a sample size of 12 subjects. To account for possible dropouts and to increase statistical power for secondary outcomes, we decided to include 20 men in this study. Owing to sex-specific differences in EE and substrate utilization, we additionally included 20 women.

Statistical analyses were carried out with GraphPad Prism (version 9.0.1). Data are presented as mean and standard deviation (SD); except in figures, where they are shown as mean and standard error of mean (SEM). Within-group differences (before vs. after) were compared by non-parametric Wilcoxon signed rank test for paired samples. Between-group differences (salt vs. placebo) were compared by Mann–Whitney U test for unpaired samples. Two-way ANOVA was used for multiple comparisons and *p* values for salt effects (salt vs. placebo) are shown in the figures. A *p* value < 0.05 indicated statistical significance.

## 3. Results

Table 1 shows the baseline characteristics of the 38 subjects who completed the study. There were no clinically relevant differences between the placebo and salt group before the intervention. Subjects reported no relevant health issues connected to the interventions. One man in the placebo group discontinued the intervention due to a common cold. One woman in the salt group could not complete the final assessment due to a migraine. Both subjects were excluded from the data analysis.

### 3.1. Primary Outcome Measure

**Figure 2:**
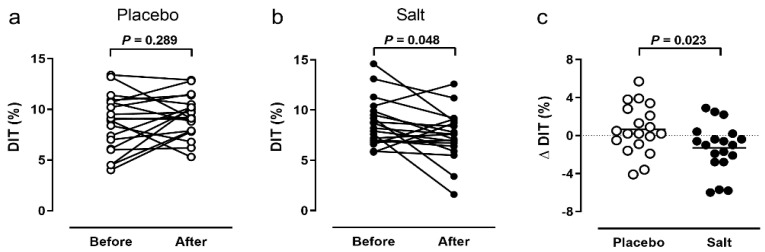
Primary outcome. Diet-induced thermogenesis (DIT) within 4 h after a 441 kcal breakfast before and after 2 weeks of placebo (*n* = 19, (**a**) or salt (*n* = 19, (**b**). Comparison of DIT changes between groups (*n* = 38, (**c**)). *P* value by Wilcoxon signed rank test (**a**,**b**) or Mann–Whitney U test (**c**).

DIT did not change in the placebo group (Figure 2a), but was significantly lower in the salt group (Figure 2b) after the intervention (placebo: 8.6 ± 2.8% vs. 9.3 ± 2.1%, change +0.6%, *p* = 0.289; salt: 8.7 ± 2.4% vs. 7.4 ± 2.5%, change −1.3%, *p* = 0.048). The change in DIT before vs. after the intervention was significantly different between the groups (*p* = 0.023, Figure 2c). We did not detect significant sex-specific differences of the primary outcome.

### 3.2. Indirect Calorimetry

Resting EE was comparable between the groups, well within normal range and not different before vs. after the intervention (placebo: 1574 ± 271 vs. 1532 ± 255 kcal/d; salt: 1592 ± 257 vs. 1586 ± 240 kcal/d). The same was true for the corresponding resting RER (placebo: 0.78 ± 0.04 vs. 0.79 ± 0.04; salt: 0.80 ± 0.06 vs. 0.82 ± 0.05).

**Figure 3:**
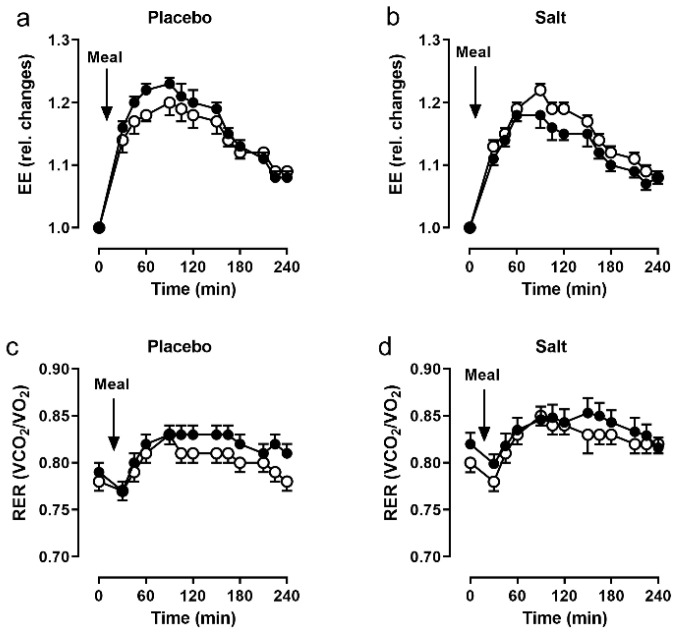
Indirect calorimetry. Energy expenditure (EE) and respiratory exchange ratio (RER) following a 441 kcal breakfast, before (open circles) and after (closed circles) 2 weeks of placebo (*n =* 19, (**a**,**c**)) and salt (*n* = 19, (**b**,**d**)). Data as mean and SEM.

In the placebo group, the increase in EE following the test meal was smaller at baseline and greater after the intervention. In the salt group, the reverse was true where the increase in EE was greater at baseline, but smaller after the intervention. However, these changes did not reach statistical significance (Figure 3a,b). There were no significant differences of RER changes after the meal between the groups (Figure 3c,d).

### 3.3. Body Composition

Body weight, fat mass, fat-free mass, body cell mass, extracellular water and total body water were not significantly different after the 2 week intervention, neither within nor between groups (Table 2).

### 3.4. Dietary Analysis

Dietary habitual salt intake before and during the intervention was comparable within and between groups (placebo: 5.5 ± 2.2 vs. 6.1 ± 2.4 g/d; salt: 6.4 ± 2.5 vs. 6.2 ± 2.2 g/d). Consequently, our intervention increased the salt intake in the salt group by approximately 100%, yielding a daily salt intake of approximately 12 g in the salt group and 6 g in the placebo group. Energy, carbohydrate, protein, fat (Table 2), and micronutrient intakes were not significantly different (data not shown). Fluid intake was comparable between groups at baseline, but tended to be higher (+470 mL) after the intervention in the salt group (*NS*; Table 2).

### 3.5. Urine Parameters

Urine volume decreased by 70 mL in the placebo group, but increased by 166 mL in the salt group (both *NS*; Table 2). Average urine sodium excretion increased by 46 mmol/L and 2.87 g/d in the salt group (Table 2). This increase in urinary sodium excretion reflects the 2.36 g/d increase in sodium intake by the salt capsules, indicating overall compliance to the intervention. Daily individual values for urine volume and urine sodium, measured from 24 h urine collections on three consecutive days, are shown in Tables S1 and S2. Urine osmolality, urea, and creatinine were not statistically different within or between groups (Table 2).

### 3.6. Cardiovascular Parameters

We measured blood pressure non-invasively during indirect calorimetry. Our relatively young, healthy cohort was normotensive and did not show clinically meaningful changes in blood pressure and other relevant cardiovascular parameters (heart rate, pulse wave velocity, and total peripheral resistance) due to the intervention (Table 2). Four subjects in the salt group had an increase in systolic blood pressure and seven had an increase in diastolic blood pressure after the intervention. This compares to 11 and 7 subjects in the placebo group with increased systolic and diastolic blood pressure, respectively.

## 4. Discussion

In this study, we aimed to confirm an observation from a previous pilot study, in which we observed a surprising decrease in DIT following a 2 week high salt intervention in healthy men. In this paper, we repeated this intervention in a larger cohort of men and women in a randomized, placebo-controlled design. We could confirm the decrease in DIT in the salt but not in the placebo group. The decrease was less pronounced than in our pilot study. This can be explained by the larger and more heterogeneous cohort, as well as by the inclusion of men and women. Interestingly, there were a few subjects whose DIT did not decrease, which could possibly indicate differently pronounced predispositions.

The decreased DIT indicates that the subjects in the salt group expended less energy to process the test meal. Our subjects had a mean caloric intake of 2230 kcal/d. Therefore, the 1.3% DIT reduction after salt would amount to 10,130 less expended kcal/year.

Since a positive energy balance leads to body weight gain throughout life, the observed salt effect could in part contribute to the progressive weight gain in Western populations. Some evidence suggests that a smaller DIT is associated with higher body mass indices [12]. However, it is not clear if the reduced DIT is cause or effect in this scenario. Our data contribute valuable evidence on this question, since we show that DIT is reduced in normal weight subjects due to the modulation of a single dietary component, which might lead to an increase in body weight in the long term.

In contrast, experimental evidence from rodents suggests that pair-fed mice under high salt conditions seem to be in a rather catabolic state, which is thought to be mediated by energy-intense urea production in liver and muscle. Urea is used for renal water conservation during high sodium excretion [13]. However, these mice were subjected to supra-physiological salt concentrations, making comparisons to the human situation difficult. In addition, high-salt-treated mice might have experienced a higher stress level due to the pair-feeding protocol, which might have confounded these results. We are not aware of any studies that found increased EE due to increased salt intake in humans.

Fluid intake and urine volume increased slightly, but non-significantly, in the salt group by 470 and 166 mL, respectively. Rakova et al. showed that water balance under high salt conditions follows rhythmical osmolyte-free water accrual and excretion [14]. These spontaneous rhythms are regulated by aldosterone and cortisone and cover about half-weekly to weekly periods, respectively [15]. We collected 24 h urine samples on three consecutive days before and after the intervention. Accordingly, a comprehensive sodium and fluid balance and a recording of these rhythms is outside the scope of our study. However, body weight and body composition measures, such as body water content, did not change, indicating that there was no relevant water retention. However, noticeable amounts of sodium can also be stored in skin and muscle without water retention, as quantified by ^23^Na MRI [16,17].

Energy, macro-, and micronutrient intake in our subjects were largely appropriate. In addition, there was no increase in energy intake due to salt as suspected previously [7,8]. Carbohydrate, fat, and protein intakes were 42–44%, 35–36%, and 16–17% of total energy intake in our cohort, respectively. The German Nutrition Society recommends more than 50% carbohydrates, less than 30% fat, and 10–11% protein intakes (according to body weight). However, the German national food consumption survey II (Nationale Verzehrsstudie II) suggests that the macronutrient pattern of our cohort is rather typical for the German population. In this representative survey, men consumed 45%, 36%, and 14% of their energy intake as carbohydrates, fats, and proteins, respectively; for women, the values were 49%, 35%, and 14%, respectively.

Both groups consumed less salt compared to a representative German population survey, which estimated that men and women consumed 10.0 and 8.4 g/d, respectively [4]. Since the reported energy intake was within normal range, a relevant under-reporting can be excluded. Our cohort was not representative of the general population in that it consisted of healthy, young-to-middle-aged academics, who generally might have healthier dietary habits. Nevertheless, it is impressive that a 2 week intervention in healthy subjects can lead to such metabolically relevant observations. The extent to which older and possibly more obese subjects might show similar or even more aggravated effects needs to be investigated in follow-up studies. Noteworthily, our evaluation as well as referenced surveys did not include added salt. Moreover, the salt content of out-of-home meals is hard to estimate.

We did not detect an increase in mean blood pressure due to increased salt intake. Twenty-five and forty-four percent of subjects had higher systolic and diastolic blood pressure, respectively, after salt intake. This is in line with studies by Weinberg et al. who reported that 25 percent of normotensive individuals were salt sensitive [18]. However, similar changes in the placebo group suggest intra-individual variations rather than actual salt effects. In our previous pilot study, we measured blood pressure in eight healthy men by 24 h ambulatory blood pressure measurement (ABPM) and found a consistent increase in nocturnal systolic (106 to 118 mmHg) and diastolic (60 to 71 mmHg) blood pressure [19]. In the present study, blood pressure was measured during indirect calorimetry. It is possible that the expected increase in blood pressure after high salt was masked by increased stress levels during the calorimetric measurements under the canopy hood compared to nocturnal sleep at home. It is known that the circumstances during blood pressure measurements profoundly influence on study outcomes [20]. Moreover, it should be noted that our study was not powered for blood pressure, but for DIT.

The strengths of our study are the confirmatory study design, the medium-sized, well-balanced cohort, and a quantitative measure of compliance via urine sodium excretion. However, there are also limitations resulting from our study design. First, we cannot determine if the decrease in DIT was due to changes in one or more of its components, i.e., EE for digestion, absorption, transport or storage of nutrients. Second, we only cover a period of two weeks. Third, this effect might be different in obese individuals who already have a reduced DIT. Fourth, all participants in our study were Caucasians, which limits the extrapolation of results to other ethnicities that might have different sensitivities to salt. Fifth, we did not control for menstrual cycle in women. However, its influence on resting EE is rather small [21]. DIT does not seem to be affected by the menstrual phase, but may be affected by oral contraceptives [22]. In our study, only two and three women in the placebo and salt group took oral contraceptives, respectively. Thus, any possible bias should have been small and distributed equally among groups. Moreover, the intervention period was exactly 2 weeks. Hence, starting the intervention with all women in the luteal phase would mean that all post-intervention measurements would fall in the follicular phase or vice versa, which would have introduced an unnecessary bias. Sixth, the DIT decrease of 1.3 percent is rather small and other influencing factors apart from increased salt intake cannot be excluded.

## 5. Conclusions

We conclude that a moderate short-term increase in salt intake affects postprandial energy metabolism in healthy men and women. We observed a relevant inter-individual variability, suggesting different susceptibilities to salt-induced health risks.

## Figures and Tables

**Table 1:** Baseline characteristics of 38 healthy volunteers before the intervention ^1^.

Characteristic	Placebo	Salt
Men/women (n)	9/10	10/9
Age (years)	29 (6)	32 (7)
Body mass index (kg/m^2^)	23 (2)	23 (3)
Waist circumference (cm)	79 (10)	79 (10)
Hip circumference (cm)	92 (9)	94 (9)
Waist/hip ratio	0.85 (0.07)	0.84 (0.08)

^1^ Data are given as means (SD).

**Table 2:** Body composition, dietary intake, urine analysis, and cardiovascular parameters of healthy volunteers before and after 2 weeks of placebo or salt ^1^.

Variable	Placebo, Before	Placebo, After	Salt, Before	Salt, After
Body weight (kg)	68 (12)	68 (13)	71 (14)	71 (14)
Fat mass (%)	25 (5)	25 (5)	23 (6)	23 (5)
Fat free mass (%)	75 (5)	75 (5)	77 (6)	77 (5)
Body cell mass (% fat free mass)	53 (3)	53 (3)	54 (3)	54 (3)
Extracellular water (L)	44 (3)	44 (3)	43 (3)	43 (3)
Total body water (%)	53 (5)	53 (5)	55 (5)	55 (4)
Energy intake (kcal)	1940 (517)	2026 (536)	2201 (481)	2251 (484)
Fat intake (% energy intake)	35 (8)	35 (7)	36 (5)	36 (4)
Carbohydrate intake (% energy intake)	44 (7)	42 (7)	43 (7)	42 (5)
Protein intake (% of energy intake)	16 (4)	16 (4)	16 (3)	17 (4)
Water intake (L)	2.70 (0.87)	2.61 (0.77)	2.78 (0.90)	3.25 (1.67)
Urine volume (mL) ^2^	2228 (763)	2158 (767)	1756 (742)	1922 (786)
Urine osmolality (mOsmol/kg) ^2^	356 (122)	450 (174)	544 (234)	583 (208)
Urine urea (g/dL) ^2^	1.15 (0.51)	1.16 (0.50)	1.46 (0.61)	1.25 (0.46)
Urine creatinine (mg/dL) ^2^	77 (27)	79 (32)	112 (67)	85 (30)
Urine sodium (mmol/L) ^2^	75 (30)	91 (34)	90 (40)	136 (56) *
Urine sodium (mmol/d) ^2^	152 (46)	183 (65)	123 (55)	248 (85) **
Urinary excretion of sodium (g/d) ^2^	3.49 (1.06)	4.21 (1.50)	2.83 (1.27)	5.70 (1.95) **
Systolic blood pressure (mmHg) ^3^	115 (7)	116 (8)	119 (7)	118 (8)
Diastolic blood pressure (mmHg) ^3^	71 (8)	71 (7)	74 (6)	74 (6)
Mean arterial pressure (mmHg) ^3^	91 (7)	92 (7)	93 (5)	94 (7)
Heart rate (1/min) ^3^	64 (10)	64 (9)	67 (9)	66 (11)
Pulse wave velocity (m/s) ^3^	5.2 (0.5)	5.2 (0.5)	5.5 (0.6)	5.5 (0.7)
Total peripheral resistance (Pa*s/m^3^) ^3^	1.14 (0.14)	1.11 (0.13)	1.12 (0.12)	1.12 (0.10)

^1^ Data are given as means (SD), *p* values by Wilcoxon signed rank test. ^2^ Mean of three consecutive 24 h urine collections per subject. ^3^ Mean of 17 measurements (every 15 min over 4 h). * *p* = 0.006, ** *p* < 0.0001.

## Data Availability

Data described in this manuscript will be made available upon request.

## Supplementary Materials

The following supporting information can be downloaded at: https://www.mdpi.com/article/10.3390/nu14020253/s1: Table S1: Individual daily values of urine volume and sodium excretion in the placebo group before and after the intervention. Table S2. Individual daily values of urine volume and sodium excretion in the salt group before and after the intervention.

## References

[1] Mente A., O’Donnell M., Rangarajan S., Dagenais G., Lear S., McQueen M., Diaz R., Avezum A., Lopez-Jaramillo P., Lanas F. (2016). Associations of urinary sodium excretion with cardiovascular events in individuals with and without hypertension: A pooled analysis of data from four studies. Lancet.

[2] Strohm D., Bechthold A., Ellinger S., Leschik-Bonnet E., Stehle P., Heseker H., German Nutrition S. (2018). Revised Reference Values for the Intake of Sodium and Chloride. Ann. Nutr. Metab..

[3] Oria M., Harrison M., Stallings V.A. (2019). Dietary Reference Intakes for Sodium and Potassium.

[4] Gosswald A., Lange M., Kamtsiuris P., Kurth B.M. (2012). DEGS: German Health Interview and Examination Survey for Adults. A nationwide cross-sectional and longitudinal study within the framework of health monitoring conducted by the Robert Koch Institute. Bundesgesundheitsblatt Gesundh. Gesundh..

[5] Ezzati M., Riboli E. (2013). Behavioral and dietary risk factors for noncommunicable diseases. N. Engl. J. Med..

[6] Global Burden of Disease Study 2017 Diet Collaborators (2019). Health effects of dietary risks in 195 countries, 1990–2017: A systematic analysis for the Global Burden of Disease Study 2017. Lancet.

[7] Grimes C.A., Riddell L.J., Campbell K.J., Nowson C.A. (2013). Dietary salt intake, sugar-sweetened beverage consumption, and obesity risk. Pediatrics.

[8] He F.J., Marrero N.M., MacGregor G.A. (2008). Salt intake is related to soft drink consumption in children and adolescents: A link to obesity?. Hypertension.

[9] Ma Y., He F.J., MacGregor G.A. (2015). High salt intake: Independent risk factor for obesity?. Hypertension.

[10] Zhou L., Stamler J., Chan Q., Van Horn L., Daviglus M.L., Dyer A.R., Miura K., Okuda N., Wu Y., Ueshima H. (2019). Salt intake and prevalence of overweight/obesity in Japan, China, the United Kingdom, and the United States: The INTERMAP Study. Am. J. Clin. Nutr..

[11] Steiniger J., Noack R. (1988). Determination of energy and substrate metabolism using indirect calorimetry. Z. Med. Lab. Diagn..

[12] Carneiro I.P., Elliott S.A., Siervo M., Padwal R., Bertoli S., Battezzati A., Prado C.M. (2016). Is Obesity Associated with Altered Energy Expenditure?. Adv. Nutr..

[13] Kitada K., Daub S., Zhang Y., Klein J.D., Nakano D., Pedchenko T., Lantier L., LaRocque L.M., Marton A., Neubert P. (2017). High salt intake reprioritizes osmolyte and energy metabolism for body fluid conservation. J. Clin. Investig..

[14] Rakova N., Kitada K., Lerchl K., Dahlmann A., Birukov A., Daub S., Kopp C., Pedchenko T., Zhang Y., Beck L. (2017). Increased salt consumption induces body water conservation and decreases fluid intake. J. Clin. Investig..

[15] Rakova N., Juttner K., Dahlmann A., Schroder A., Linz P., Kopp C., Rauh M., Goller U., Beck L., Agureev A. (2013). Long-term space flight simulation reveals infradian rhythmicity in human Na(+) balance. Cell Metab..

[16] Linz P., Santoro D., Renz W., Rieger J., Ruehle A., Ruff J., Deimling M., Rakova N., Muller D.N., Luft F.C. (2015). Skin sodium measured with (2)(3)Na MRI at 7.0 T. NMR Biomed..

[17] Titze J. (2014). Sodium balance is not just a renal affair. Curr. Opin. Nephrol. Hypertens..

[18] Weinberger M.H. (1991). Salt sensitivity as a predictor of hypertension. Am. J. Hypertens..

[19] Wilck N., Matus M.G., Kearney S.M., Olesen S.W., Forslund K., Bartolomaeus H., Haase S., Mahler A., Balogh A., Marko L. (2017). Salt-responsive gut commensal modulates TH17 axis and disease. Nature.

[20] Group S.R., Wright J.T., Williamson J.D., Whelton P.K., Snyder J.K., Sink K.M., Rocco M.V., Reboussin D.M., Rahman M., Oparil S. (2015). A Randomized Trial of Intensive versus Standard Blood-Pressure Control. N. Engl. J. Med..

[21] Benton M.J., Hutchins A.M., Dawes J.J. (2020). Effect of menstrual cycle on resting metabolism: A systematic review and meta-analysis. PLoS ONE.

[22] Duhita M.R., Schutz Y., Montani J.P., Dulloo A.G., Miles-Chan J.L. (2017). Oral Contraceptive Pill Alters Acute Dietary Protein-Induced Thermogenesis in Young Women. Obesity.

