# Supplementary material for: Increased Salt Intake Decreases Diet-Induced Thermogenesis in Healthy Volunteers: A Randomized Placebo-Controlled Study
Source: Nutrients. 2022 Jan 7;14(2):253. doi: 10.3390/nu14020253 (PMC8779306; doi:10.3390/nu14020253)
Supplement: Supplementary file 1 [file nutrients-14-00253-s001.zip › nutrients-1513419-supplementary.pdf]

Supplementary Table S1. Individual daily values of urine volume and sodium excretion in the placebo group before and after the intervention.

**Placebo, before**		**M1**	**M2**	**M3**	**M4**	**M5**	**M6**	**M7**	**M8**	**M9**	**W1**	**W2**	**W3**	**W4**	**W5**	**W6**	**W7**	**W8**	**W9**	**W10**
**Urine volume (mL)**	**d1**	1800	2100	1000	ND	1400	1820	1000	3700	2480	3000	4660	2400	2400	2600	2800	580	2270	1100	1600
	**d2**	2100	1500	700	2700	2000	1850	1600	2150	1620	3650	4400	1500	1720	1500	2600	2480	2000	1720	2800
	**d3**	2700	1550	2780	3450	1530	1800	900	3200	2050	5400	2500	2250	1500	1680	2100	1800	1500	2770	2520
	**d 1-3**	2200	1717	1493	3075	1643	1823	1167	3017	2050	4017	3853	2050	1873	1927	2500	1620	1923	1863	2307
**Urine sodium (mmol/L)**	**d1**	133	83	181	ND	83	142	80	71	71	36	44	47	55	28	52	81	121	96	118
	**d2**	130	96	164	34	116	107	113	88	104	64	32	51	44	ND	44	88	122	66	76
	**d3**	73	115	72	48	68	42	67	71	92	31	29	45	34	47	55	46	92	73	59
	**d 1-3**	112	98	139	41	89	97	87	77	89	44	35	48	44	38	50	72	112	78	84
**UNaV (g/d)**	**d1**	5,5	4,0	4,2	ND	2,7	5,9	1,8	6,0	4,0	2,5	4,7	2,6	3,0	1,7	3,3	1,1	6,3	2,4	4,3
	**d2**	6,3	3,3	2,6	2,1	5,3	4,6	4,2	4,3	3,9	5,4	3,2	1,8	1,7	ND	2,6	5,0	5,6	2,6	4,9
	**d3**	4,5	4,1	4,6	3,8	2,4	1,7	1,4	5,2	4,3	3,8	1,7	2,3	1,2	1,8	2,7	1,9	3,2	4,6	3,4
	**d 1-3**	5,4	3,8	3,8	3,0	3,5	4,1	2,5	5,2	4,1	3,9	3,2	2,2	2,0	1,7	2,9	2,7	5,0	3,2	4,2
**Placebo, after**		**M1**	**M2**	**M3**	**M4**	**M5**	**M6**	**M7**	**M8**	**M9**	**W1**	**W2**	**W3**	**W4**	**W5**	**W6**	**W7**	**W8**	**W9**	**W10**
**Urine volume (mL)**	**d1**	1350	1960	1630	2150	1100	2400	1520	2700	2900	4500	4500	1980	2100	2000	3100	2550	900	1280	3350
	**d2**	1630	1400	2250	3200	1350	1340	1980	2100	1850	2650	4300	2200	1280	2280	3400	2420	1400	650	1400
	**d3**	2210	1000	2700	2550	1980	1600	2250	1600	1200	3900	2600	2700	1900	2580	2800	2600	1400	860	800
	**d 1-3**	1730	1453	2193	2633	1477	1780	1917	2133	1983	3683	3800	2293	1760	2287	3100	2523	1233	930	1850
**Urine sodium (mmol/L)**	**d1**	119	106	49	94	115	141	87	68	87	61	23	78	54	25	25	67	130	49	150
	**d2**	199	66	114	122	66	159	107	115	137	42	34	67	163	67	103	55	135	97	103
	**d3**	149	79	136	100	76	107	96	86	167	32	20	51	104	71	85	65	117	71	111
	**d 1-3**	156	84	100	111	86	136	97	90	130	45	26	65	107	48	54	62	127	72	121
**UNaV (g/d)**	**d1**	3,7	4,8	1,8	4,7	2,9	7,8	3,0	4,2	5,8	6,3	2,4	3,6	2,6	1,1	1,8	3,9	2,7	1,4	11,6
	**d2**	7,5	2,1	5,9	9,0	2,0	4,9	4,9	5,6	5,8	2,6	3,4	3,4	4,8	3,5	8,1	3,1	4,3	1,4	3,3
	**d3**	7,6	1,8	8,4	5,9	3,5	3,9	5,0	3,2	4,6	2,9	1,2	3,2	4,5	4,2	5,5	3,9	3,8	1,4	2,0
	**d 1-3**	6,2	2,9	5,4	7,4	2,8	5,5	4,3	4,3	5,4	3,9	2,3	3,4	4,0	3,0	5,1	3,6	3,6	1,4	5,6

D, day; M, man; ND, not determined; W, woman

Supplementary Table S2. Individual daily values of urine volume and sodium excretion in the salt group before and after the intervention.

**Salt, before**		**M10**	**M11**	**M12**	**M13**	**M14**	**M15**	**M16**	**M17**	**M18**	**M19**	**W11**	**W12**	**W13**	**W14**	**W15**	**W16**	**W17**	**W18**	**W19**
**Urine volume (mL)**	**d1**	300	2100	1200	1400	3200	2620	1500	2020	1280	900	700	2500	740	2000	1650	2100	1200	1050	2900
	**d2**	200	820	2250	1800	3200	3050	2400	1320	1100	800	1850	3500	1430	2200	2000	2600	1580	1300	2930
	**d3**	980	1200	1980	2300	4000	2180	850	2100	1000	800	1200	1800	1410	2800	2100	2000	1200	700	1800
	**d 1-3**	493	1373	1810	1833	3467	2617	1583	1813	1127	833	1250	2600	1193	2333	1917	2233	1327	1017	2543
**Urine sodium (mmol/L)**	**d1**	80	46	196	83	57	42	149	90	117	159	80	52	122	37	77	62	43	114	34
	**d2**	83	117	116	87	43	38	99	170	157	143	63	57	131	39	71	80	50	131	28
	**d3**	50	124	94	76	65	73	119	140	200	152	77	67	75	56	67	64	64	210	39
	**d 1-3**	71	96	135	82	55	51	122	133	158	151	73	59	109	44	72	69	52	152	34
**UNaV (g/d)**	**d1**	0,6	2,2	5,4	2,7	4,2	2,5	5,1	4,2	3,4	3,3	1,3	3,0	2,1	1,7	2,9	3,0	1,2	2,8	2,3
	**d2**	0,4	2,2	6,0	3,6	3,2	2,7	5,5	5,2	4,0	2,6	2,7	4,6	4,3	2,0	3,3	4,8	1,8	3,9	1,9
	**d3**	1,1	3,4	4,3	4,0	6,0	3,7	2,3	6,8	4,6	2,8	2,1	2,8	2,4	3,6	3,2	2,9	1,8	3,4	1,6
	**d 1-3**	0,7	2,6	5,2	3,4	4,4	3,0	4,3	5,4	4,0	2,9	2,0	3,4	2,9	2,4	3,1	3,6	1,6	3,3	1,9
**Salt, after**		**M10**	**M11**	**M12**	**M13**	**M14**	**M15**	**M16**	**M17**	**M18**	**M19**	**W11**	**W12**	**W13**	**W14**	**W15**	**W16**	**W17**	**W18**	**W19**
**Urine volume (mL)**	**d1**	1100	1340	2200	1900	3800	3080	1620	1200	1500	710	1880	2700	1800	ND	2880	1410	2150	1280	2050
	**d2**	1720	1000	1700	2100	3700	3060	1700	1460	1700	1350	1250	3700	2500	3200	2500	2500	2100	1240	4100
	**d3**	900	1480	1000	3000	2600	2080	1900	1980	1400	1010	2200	2100	2400	3600	2680	1690	1400	1120	1000
	**d 1-3**	1240	1273	1633	2333	3367	2740	1740	1547	1533	1023	1777	2833	2233	3400	2687	1867	1883	1213	2383
**Urine sodium (mmol/L)**	**d1**	164	268	144	142	76	83	231	105	240	99	127	102	131	ND	94	108	114	232	71
	**d2**	127	276	139	94	48	62	185	189	242	175	100	86	138	86	98	130	98	207	79
	**d3**	174	208	111	109	73	89	173	122	238	252	147	100	132	50	96	106	108	197	106
	**d 1-3**	155	251	131	115	66	78	196	139	240	175	125	96	134	68	96	115	107	212	85
**UNaV (g/d)**	**d1**	4,1	8,3	7,3	6,2	6,6	5,9	8,6	2,9	8,3	1,6	5,5	6,3	5,4	ND	6,2	3,5	5,6	6,8	3,3
	**d2**	5,0	6,3	5,4	4,5	4,1	4,4	7,2	6,3	9,5	5,4	2,9	7,3	7,9	6,3	5,6	7,5	4,7	5,9	7,4
	**d3**	3,6	7,1	2,6	7,5	4,4	4,3	7,6	5,6	7,7	5,9	7,4	4,8	7,3	4,1	5,9	4,1	3,5	5,1	2,4
	**d 1-3**	4,3	7,2	5,1	6,1	5,0	4,8	7,8	4,9	8,5	4,3	5,3	6,2	6,9	5,2	5,9	5,0	4,6	5,9	4,4

D, day; M, man; W, woman
